# Cold atmospheric plasma: a sustainable approach to inactivating viruses, bacteria, and protozoa with remediation of organic pollutants in river water and wastewater

**DOI:** 10.1007/s11356-023-30298-x

**Published:** 2023-11-01

**Authors:** Ibrahim Ahmed Hamza, Amer S. El-Kalliny, Sherif Abd‑Elmaksoud, Mohamed A. Marouf, Mahmoud S. Abdel‑Wahed, Mohamed Azab El-Liethy, Mohamed Mokhtar Hefny

**Affiliations:** 1 Water Pollution Research Department, National Research Centre, 33 El Buhouth St., Dokki, Giza, 12622 Egypt; 2 Engineering Mathematics and Physics Department, Faculty of Engineering and Technology, Future University in Egypt, Cairo, Egypt

**Keywords:** Cold atmospheric plasma, Viruses, Bacteria, Protozoa, River water, Wastewater

## Abstract

Innovative technologies are needed to enhance access to clean water and avoid waterborne diseases. We investigated the performance of cold atmospheric plasma (CAP), a clean and sustainable approach for microbial inactivation and total organic carbon (TOC) degradation in environmental water. Water matrices played a crucial role in the performance of CAP efficacy; for example, complete removal of ɸX174 from dH_2_O required 1 min of treatment, while ɸX174 reductions of ~ 2log_10_ and 4log_10_ were obtained after 10 min of CAP exposure in river water and wastewater samples, respectively. Similarly, after 10 min of CAP treatment, bacterial concentrations decreased by 3 log_10_ and 4 log_10_, in river and wastewater samples, respectively. In contrast, after 30 s of contact time, a 4 log_10_ reduction of bacteria was accomplished in dH_2_O. Complete removal of *Acanthamoeba* from dH_2_O was found after 30 min of CAP treatment, whereas it was not removed from surface water or wastewater at the same exposure time. Additionally, the approach successfully reduced TOC, and the degradation kinetics of TOC were represented by pseudo-first-order. CAP showed higher rates of TOC degradation in the final effluent of the wastewater treatment plant compared to surface water. The difference in CAP performance between river water and wastewater could be attributed to the bulk structure of humic acids in river water compared to small organic byproducts in the final effluent of WWTP. Overall, the findings reported here support the idea that CAP holds promise as a sustainable solution for controlling pathogens, removing organic water pollution, and integrating with traditional purification processes. Low-cost systems may advance CAP technology and increase its widespread use.

## Introduction

Water contamination has become an important global concern due to population growth, climate change, industrial expansion, and agricultural expansion. Water pollution and poor sanitation have been associated with the transmission of diseases such as cholera, diarrhea, hepatitis A, typhoid, and poliomyelitis (WHO 2022a).

According to the WHO, 829,000 people die every year from diarrheal disease as a result of poor hand hygiene, poor sanitation, and unsafe drinking water. However, diarrhea is generally avoidable, and if these risk factors were managed, 297,000 infant deaths under the age of five every year could be averted. The discharge of untreated and/or poorly treated wastewater typically has an impact on downstream waterbodies and pollutes groundwater (WHO 2022b).

Conventional treatment methods such as chlorination, UV irradiation, ozonation, and filtration have been widely researched and proven to be successful in removing a wide spectrum of microorganisms (Abdo et al. 2022; Hamza and Abd-Elmaksoud 2023; Shi et al. 2021). These approaches are extensively acknowledged and applied by water treatment plants and have well-established regulatory criteria. However, the technologies used to treat water currently have some limitations. For example, chlorine can react with organic substances, resulting in the production of harmful reactive chlorinated organic compounds for human health. The operation and maintenance of other disinfection agents, including ozone and ultraviolet irradiation, are complex and cannot provide long-term protection when it comes to distribution systems. On the other hand, conventional technologies cannot be applied to treat persistent organic compounds that are soluble in water like pharmaceuticals, halogenated hydrocarbons, aromatic chemicals, and pesticides (Arbuckle et al. 2002).

Advanced oxidation processes (AOPs), including photocatalysis, ozonation, electrochemical treatments, non-thermal processes, Fenton, and photo-Fenton processes, represent emerging and innovative methods for water decontamination. The effectiveness of AOPs in reducing complex and non-biodegradable compounds has attracted significant research attention. In principle, these processes involve introducing electrical, radiation, and/or chemical energy into the reaction zone, which results in the degradation of complex chemicals (Graumans et al. 2020; Mahmoud and El-Liethy 2022). Also, AOPs can be used along with conventional methods such as filtering to remove pollutants that are resistant to chlorine (e.g., bacterial spores and protozoa).

CAP is considered one of the most effective AOP which generates high densities of reactive oxygen species (ROS) and reactive nitrogen species (RNS) due to the high energetic electrons in CAP (Abdel-Wahed et al. 2022; Adamovich et al. 2022). Several reactive species may be formed and categorized into two groups: long-lived species like H_2_O_2_, NO_2_, and NO_3_, and short-lived species like O, ·NO, ·OH, O_2_^·−^, and ^1^O_2_. Reactive species have variable reaction rate constants in the gas and liquid phase, and they can be transformed into each other (Fridman et al. 2008). Reactive oxygen and nitrogen species (RONS) are the main components in CAP responsible for the anti-microbial effect, whereas UV photons have just a minimal impact (Nicol et al. 2020).

Generally, plasma exhibits quasineutrality, with relatively similar electrons and ions densities but different temperatures and is typically classified as thermal or non-thermal plasma. Thermal plasma is in thermal equilibrium, which indicates the temperatures of electrons and heavy particles are rather constant, which typically vary in the thousands of Kelvins. CAP are partly ionized gases with electron temperatures in the tens of thousands of degrees Celsius, whereas heavier particles (ions and neutrals) have substantially lower temperatures (Brany et al. 2020; El-Kalliny et al. 2021).

A number of variables can influence the effectiveness of a CAP treatment, such as the gas type, gas flow rate, water flow rate, voltage, frequency, and mode of exposure. Furthermore, internal factors such as microbial species and the structure of microbial cells have a significant impact on reaction mechanisms, kinetics, and performance, as do external factors such as pH, conductivity, and relative humidity (Murugesan et al. 2020a; Murugesan et al. 2020b).

Introducing CAP into the industry is of great importance, but a lot of challenges should be solved before achieving that. The ideal plasma system that can cover all the above-mentioned critical variables to implement this technology in the industry on a large scale has not been achieved yet. However, many developments based on pollutant degradation and consumed energy have been performed recently to achieve this. Many important perspectives should be considered for scaling up this technology such as maximization in the production of available reactive species, the large contact area between plasma and pollutants, and reaching the optimum treatment conditions with respect to energy and pollutant degradation efficiency. The sustainability of the treatment process indeed depends on the operation cost in terms of energy consumption, and recently a lot of efforts have been dedicated to decrease that cost, making plasma technology promising in water treatment. For example, Gerrity et al., using a pilot-scale cold plasma, reported that the electrical energy per order (EEO), defined as the amount of electrical energy in kWh needed to reduce the concentration of a contaminant by one order of magnitude in 1 m^3^ of polluted water, is less than 10 in the case of cold plasma treatment, while it is more than 100 in the case of biodegradation photocatalysis treatment. The energy supplied in any plasma system can be expressed by the applied voltage, power, and frequency, where cold plasma can be ignited with a wide range of frequency spectrum such as DC, AC (as in the case of the present study and many others) till nanosecond pulsed systems, which affects by its role the energy efficiency. For example, a significant enhancement in energy efficiency from 1 to 3 order of magnitude was observed when changing the ignition method from sinusoidal and/or microsecond pulsed high voltage to nanosecond pulsed systems. Accordingly, more efforts should be made to scale up plasma systems for water treatment, and more reference systems should be designed to help researchers around the world make a right comparison between systems and to bring plasma technology into water treatment plants (Aggelopoulos 2022; Gerrity et al. 2010).

Different studies have investigated the disinfection properties of CAP against both Gram-positive and Gram-negative bacteria. (Abreu et al. 2013; Younis et al. 2020). The reactive species can either interact with the bacterial outer membrane or quickly enter the cells and cause rupture of the bacterial cells (Abdel-Wahed et al. 2022). Additionally, there is a different sensitivity to CAP treatment between Gram-negative and Gram-positive bacteria. Typically, Gram-positive bacteria are more resistant to CAP compared to Gram-negative bacteria (Mai-Prochnow et al. 2014). CAP has also been proven to have the potential to treat bacteria in biofilms; however, more CAP exposure time is required for the complete removal of bacterial biofilm (Mai-Prochnow et al. 2014; Rao et al. 2020).

On the other hand, limited data on viral removal by CAP is available, and most studies have been conducted using dH_2_O. Norovirus, adenovirus, and hepatitis A viruses are common enteric viruses that have been treated with CAP (Abdel-Wahed et al. 2022; Aboubakr et al. 2016; Chen et al. 2020; Guo et al. 2018). CAP has successfully treated enteric viruses in aqueous solutions as well as in other liquid media. Also, CAP was applied to bacteriophages such as MS2, T4, and ɸX174 either through direct treatment or indirect exposure (Guo et al. 2018). All three types of bacteriophages were effectively inactivated using both methods. However, a shorter treatment time was sufficient to inactivate ɸX174 and MS2 compared to T4.

It is worth noting that there is no data available on the effect of CAP on *Acanthamoeba*. Whereas, others have tested the effect of CAP on protozoa using *Cryptosporidium parvum* as a model on cilantro-contaminated leaves (Craighead et al. 2020).

Here, we examined the effect of CAP on bacteria, viruses, and protozoa in different environmental water matrices. Additionally, the efficacy of CAP treatment was determined by the removal of total organic carbon (TOC) from the environmental samples. We used a high voltage AC power supply as a power source of our CAP with frequency in range of kHz as it will be discussed in detail in the manuscript, there are many CAP sources based on AC-driven sources with different configurations such as one atmosphere uniform glow discharge plasma (OAUGDP) (Park et al. 2012), the parallel plate reactor (Montie et al. 2000), the remote exposure reactor (Park et al. 2012), the two-rod configuration (Reece Roth et al. 2005), plasma jet with the powered electrode at the center of the tube (Perni et al. 2007), plasma jet with ring-shaped powered electrode (Deng et al. 2007), plasma jet with floating electrode inside a tube (Park et al. 2011), pin-to-pin electrodes (Sun et al. 2023), and pin-to-line (as in the case of our system configuration) (Abdel-Wahed et al. 2022; El-Kalliny et al. 2021; Park and Cha 2023).

## Material and methods

### The pin cold atmospheric plasma system

**Fig. 1:**
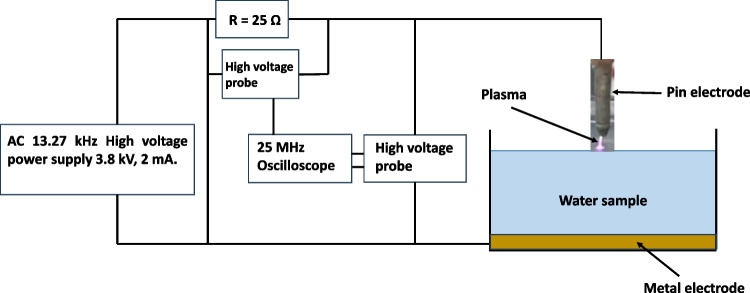
A schematic diagram of the plasma treatment system

A plasma reactor with a high-voltage AC power supply capable of supplying an input voltage ranging from 0 to 18 V and an output voltage of up to 10 kV was used. The positive terminal of the power supply was connected to the pin electrode above the water surface, while the negative terminal was connected to the platinum metal electrode immersed in the water sample. The water sample of 10 mL was treated in a Petri dish (inner diameter of 5 cm, water thickness of 3.6 mm, and gap distance between the surface of the water and plasma of 1 mm). The Petri dish was placed in contact with ambient air. To measure the plasma voltage during treatment, the plasma electrodes were outfitted with a high-voltage probe (P5101) with a division ratio of 1/1000. Moreover, the plasma current was obtained from the measurements of the voltage across a high-voltage resistor 25 Ω, which also was used to protect the electric circuit from high short-circuit current, and then divided by 25 Ω to get the current value (Allam et al. 2016), see Fig. 1, and an oscilloscope (25 MHz) was used to observe the electrical characteristics; more details about the reactor setup can be found in Fig. 1.

### Water samples

River Nile water samples were collected from the mainstream of the river in a one-liter wide-mouth sterile bottle. The final wastewater effluent sample was collected from the Zenin wastewater treatment plant (WWTP), Cairo. The collected samples were transferred in an icebox to the laboratory within 2 h. Some physicochemical parameters, including pH, electrical conductivity (EC), and total dissolved solids (TDS), were determined in the collected samples before and after CAP treatment according to APHA (2017). Cationic and anionic species were also measured by ion chromatography (Metrohm Ommnis Titrator). The degradation reaction of organic pollutants in wastewater and river Nile water was monitored by measuring TOC using the Analytik Jena, multi N/C 3100 TOC Analyzer. Moreover, 10 mL of each water type was used for CAP treatment after spiking with microbes under study.

###  Inactivation of viruses by CAP

#### Somatic coliphages

ɸX174 was exposed to plasma at different time intervals (0, 2, 5 and 10 min) in dH_2_O, river water and effluent of WWTP. ɸX174 was enumerated in water samples by a double agar layer plaque test according to ISO (2002). Briefly, *Escherichia coli* DSM 13127 grown on tryptone broth was used as a host. Coliphage was propagated in tryptone broth with a fresh (4 h-old) *E. coli* culture to generate a stock suspension. The quantity of infectious somatic coliphage was calculated as follows: 1 mL of exponentially growing host strain bacteria, 100 µL of treated or untreated water samples, and 3 mL of melting tryptone top agar were mixed and poured over modified tryptone bottom agar plates. Plaques were enumerated and expressed as PFU/mL within 3–5 h of incubation at 37 °C. Negative and positive controls were included in each test to ensure proper *E. coli* growth and coliphage detection.

#### Rotavirus

Rotavirus (RoV) SA11 was used as a model for RNA enteric viruses. Rotavirus was activated with 10 µg/mL trypsin before being propagated on MA-104 cell line monolayers using 2% FBS Dulbecco’s modified Eagle medium and incubated for 5 days at 37 °C in a 5% CO_2_ atmosphere. To eliminate cell debris, RoV-containing cell culture harvests of MA-104 cells was centrifuged for 5 min at 3000 g, and the supernatant was used as a stock suspension after filtration through a 0.22 µm membrane. RoV was subjected to different doses of plasma at different time intervals (0, 2, 5 and 10 min) in dH_2_O, river water and effluent of WWTP.

For quantitative measurement of RoV in the treated and untreated water samples, MA-104 cells were seeded into 24-well tissue culture plates (Nunclon, Roskilde, Denmark) and incubated further until they reached a confluent density of around 5.0 × 10^4^ cells per well. The cells were then infected with 100 µL of a serial dilution of CAP-treated and non-treated water samples previously prepared in culture media without FBS. After 60 min of incubation, 900 µL of DMEM without FBS was added and cultured for 3–5 days at 37 °C under 5% CO_2_. Cells were microscopically examined, and the cytopathic effect was recorded. The viral concentration was indicated as TCID50/mL, which corresponded to 50% of wells exhibiting CPE at a given sample dilution. Integrated cell culture RT-qPCR (ICC-RT-qPCR) and RT-qPCR of RoV were used to compare the effect of CAP in dH_2_O on both viral nucleic acid and infectivity using the RoV primers and qPCR conditions as described in Hamza et al. (2009). 

### Inactivation of bacteria by CAP

*E. coli* ATCC 25922 was employed as a bacterial contamination model. In a falcon tube containing 50 mL of tryptic soya broth (Merck, Germany), the stock of *E. coli* strain was injected. The inoculated tube was incubated at 37 °C for 18–24 h before being centrifuged at 4000 rpm for 20 min. To remove any enrichment broth residue, the supernatant was removed, and the pellet was rinsed three times with sterile distilled water. The *E. coli* cells were then resuspended in 25 mL of sterile distilled water.

The prepared fresh *E coli* (~ 10^7^ CFU/mL) was spiked in 10 mL of sterile distilled water (dH_2_O). The *E. coli* count was determined before and after treatment of environmental samples in a sterile petri dish. The samples were collected at zero-time exposure as an initial *E. coli* count, and after that at 2, 5, and 10 min. The *E. coli* density in the tested sterile distilled water was determined using the pour plate method on plate count agar (Merck, Germany) (APHA (2017). On the other hand, *E. coli* in the environmental water samples (river and effluent of WWTP samples) was determined on selective medium for *E. coli* namely HiCrome ECC agar (HiMedia, India) using spread plate method. All inoculated plates were incubated at 37 °C for 24 h. The *E. coli* colonies on agar plates were counted using the colony counter (Stuart, Germany). *E. coli* colonies on the surface of HiCrome ECC agar appeared in blue to purple color.

### Inactivation of protozoa by CAP

#### Isolation of *Acanthamoeba* spp

*Acanthamoeba* used in the current research was isolated from the Egyptian aquatic environment and identified as *Acanthamoeba* T4. Briefly, using a stainless-steel filter holder attached to a suction pump, 1 L of river water sample was filtered via a nitrocellulose membrane (0.45-µm pore size and 47-mm diameter). Filtration was stopped just before dryness of the membrane (HPA 2014). After the filtration process, the membrane was inverted face-to-face on the surface of a non-nutrient agar plate seeded with heat-killed *E. coli*. The plates were incubated for 7 days and examined daily under the inverted microscope (Olympus CXK 41, Japan). Isolated and purified freshwater *Acanthamoeba* was identified based on trophozoite and cyst characteristics (Page 1988). Three sequential applications of freezing and thawing in liquid nitrogen were used to disrupt the wall of *Acanthamoeba* cysts, followed by a 2-min incubation in a water bath at 100 °C. The DNA of *Acanthamoeba* was then extracted using the QIAamp DNA Stool Mini Kit (Qiagen, Valencia, CA). PCR was used to amplify a 450–500 bp DNA fragment using general primers (JDP1 and JDP2) in order to identify *Acanthamoeba* spp. according to Schroeder et al. (2001). The positive PCR products were purified using the GeneJET PCR Purification Kit (Thermo Scientific, USA), according to the manufacturer’s instructions. Nucleotide sequences were analyzed and compared to the data on GenBank. The sequence of the isolated strain has been deposited in GenBank with accession number OR143778.

####  Exposure of Acanthamoeba to CAP

*Acanthamoeba* was subjected to different doses of plasma at different time intervals (5, 10, 15, and 30 min), followed by cultivation according to the standard methods (HPA 2014). In brief, 10 mL of each spiked water sample was concentrated by using a nitrocellulose membrane filter (0.45-µm pore size and 47-mm diameter), and cultivated as described above. Treatment experiments have been conducted in triplicate. In order to investigate the effect of CAP on *Acanthamoeba* DNA, the treated and non-treated *Acanthamoeba* cysts were disrupted by three consecutive cycles of freezing and thawing in liquid nitrogen, followed by incubation in a water bath at 100 °C for 2 min. *Acanthamoeba* DNA was then extracted using the QIAamp DNA Stool Mini Kit (Qiagen, Valencia, CA). PCR was performed to amplify 450–500 bp DNA fragment using JDP1 and JDP2 generic primers (Schroeder et al. 2001). SYBR Green PCR was used to compare the CT values of treated and no treated samples.

## Results and discussion

**Fig. 2:**
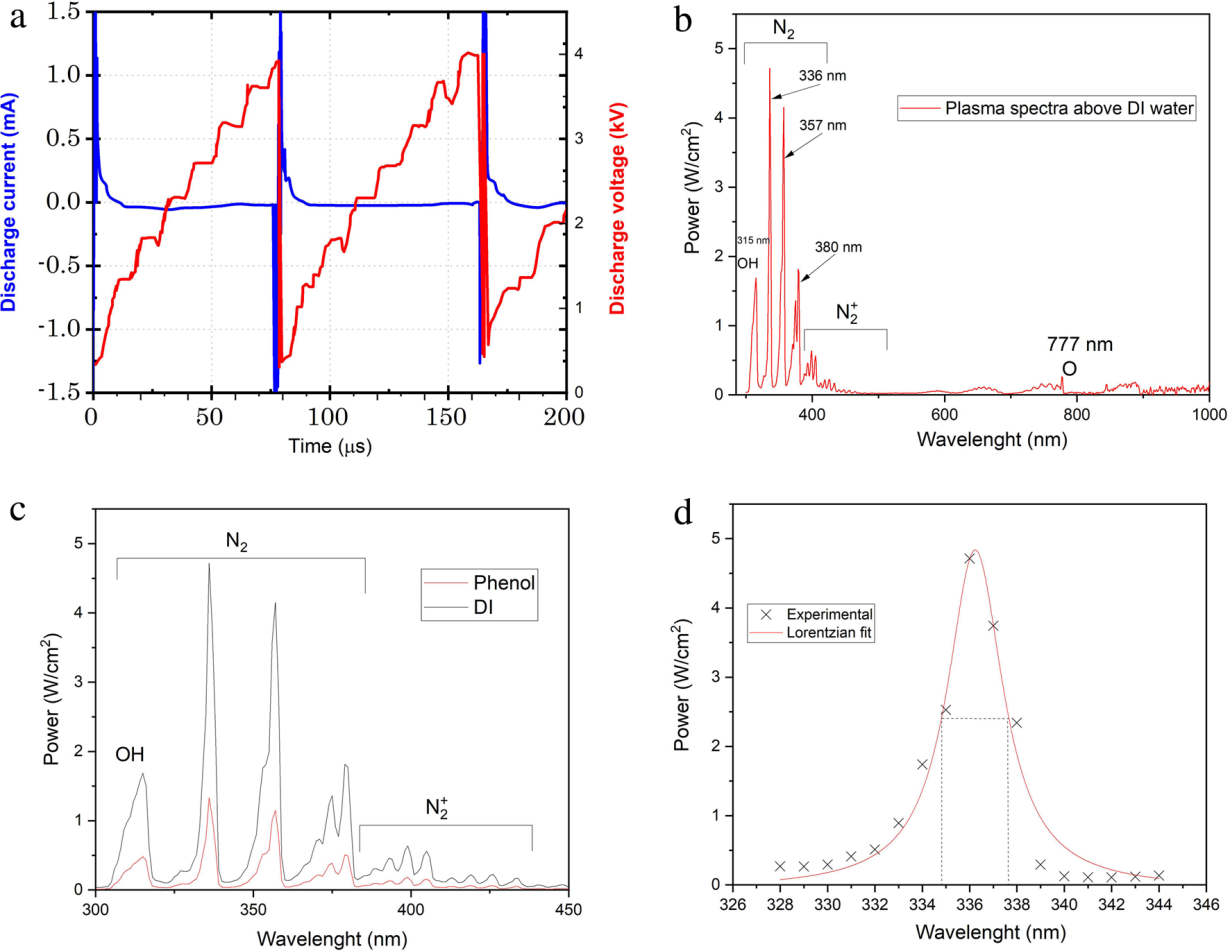
**a** Plasma voltage and current waveforms, **b** the OES of plasma above dH_2_O, **c** the difference between OES above two different media (dH_2_O and phenol), and **d** the Lorentzian fitting of N_2_ 336 nm line profile for measuring electron density using Stark broadening method

The waveforms of plasma discharge voltage and current during the treatment can be seen in Fig. 2a. The plasma voltage was around 3.8 kV (peak-to-peak), and the plasma current was around 2 mA at plasma frequency of 13.27 kHz. The consumed power (P) can be obtained from the following equation:

1 $$P= \frac{1}{T} \int {V}_{d}\left(t\right) \cdot i\left(t\right) dt$$

where *T* is the periodic time of the discharge voltage, *V*_*d*_(*t*) is the discharge voltage, and *i*(*t*) is the discharge current (Adhami Sayad Mahaleh et al. 2023; Ahmed et al. 2014). Then, the energy can be estimated through multiplying the consumed power by the periodic time. Following Eq. (1), it was found that the consumed power is 1.99 W, and the mean consumed energy per voltage period is 1.5 × 10^−4^ J. The optical emission spectra (OES) of plasma were measured above dH_2_O water using a spectral measurement system (SMS-500) with a wavelength accuracy of ± 0.25 nm (see Fig. 2b). Several lines can be observed in Fig. 2b, where OH can be seen between 306 and 317 nm with a maximum peak at 315 nm; N_2_ can be seen from 306 to 380 nm with peaks at 336, 357, and 380 nm.${N}_{2}^{+}$ lines can also be detected between 391 and 470 nm. The atomic oxygen line at 777 nm is also observed. The OES of plasma above dH_2_O were compared with the OES above phenol (as a model compound of organic pollutants in water) in a previous work (Abdel-Wahed et al. 2022), where the spectra were higher in the case of dH_2_O than in the case of phenol, and this can be attributed to the difference in diffusivity and reactivity between the plasma-generated reactive species and the treated media (see Fig. 2c), or in other words: the phenol consumes the ROS and RNS much higher than the dH_2_O (Farhan Hanafi and Sapawe 2020). The electron density (*n*_*e*_) is one of the most important plasma parameters, and excited electrons are responsible for the induced chemistry in air and liquid as well. We used the Stark broadening method to estimate *n*_*e*_ of our system, see Fig. 2d. *n*_*e*_ can be estimated from the OES of plasma by choosing one of the spectral lines (we choose here the N_2_ 336 nm line) and calculating the full width at half maximum (FWHM) of the Stark broadening *λ*_Stark_, and then substituting in the following equation:

2 $${\lambda }_{Stark}=2 \times {10}^{-11} {n}_{e}^{2/3}$$

The electron density was found (using Eq. (2_) to be approximately 5 × 10^16^ cm^−3^, where Fig. 2c shows Stark broadening and the Lorentzian fitting of the experimental data with ${\lambda }_{Stark}\approx 2.81 \mathrm{nm}.$ It is noteworthy that the electron temperature of this system was also estimated to be 6191 °K, in our previous work (Abdel-Wahed et al. 2022).

The performance of CAP was investigated in river water samples and the final effluents of the WWTP. Measuring TOC degradation in river water or wastewater is a useful tool for assessing the overall performance and efficiency of water or wastewater treatment facilities as well as an indication of water quality. It also allows for the evaluation of certain treatment methods or technologies in terms of their capacity to remove organic chemicals, process optimization and control, and regulatory compliance.

**Fig. 3:**
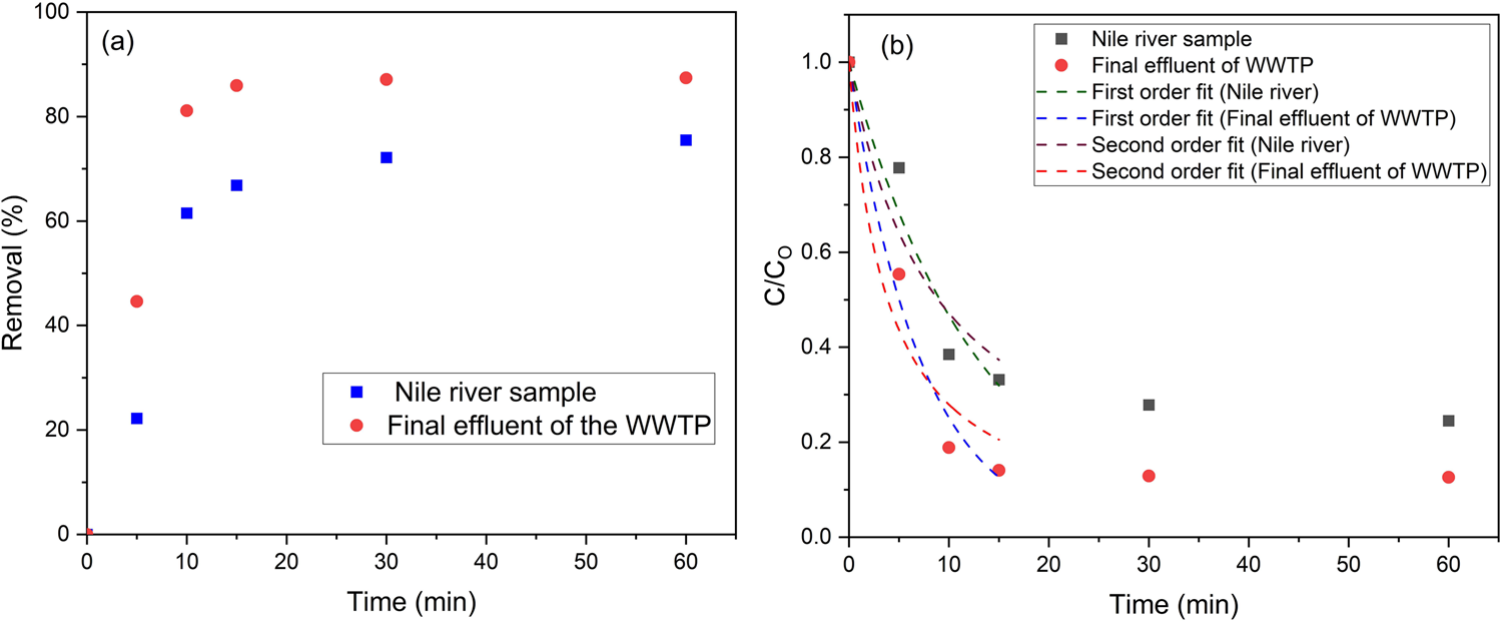
**a** The TOC percentage of removal by CAP treatment in the river and final effluent of WWTP samples. Checking of order kinetics for TOC removal of the river sample (**b**) and final effluent of the WWTP. Points and dashed lines represent the experimental values and curve fittings of the order kinetics of TOC removal, respectively

CAP treatment showed high efficiency for mineralization, as within 15 min there were 67 and 86% TOC removal for the Nile River water sample and final effluent of WWTP, respectively (Fig. 3a). This could be due to the bulk structure of humic acids (HA) in the river sample relative to small organic byproducts in the final effluent of the WWTP (Abdel-Wahed et al. 2021). This is also reflected in the slower rate of TOC degradation in the case of the river sample and low values for the rate constant (${k}_{1}$) (Table 1). Figure 3b shows non-linear regression for pseudo-first-order (Eq. (3)) and pseudo-second-order (Eq. (3)) kinetics. The non-linear equation forms for both orders are as follows (Perrin 2017):

3 $$C={C}_{o}{e}^{-{k}_{1}t},$$

4 $$C=\frac{{C}_{o}}{1+2{C}_{o}{k}_{2}t},$$

where, ${C}_{o}$ (mg/L) is the concentration of the TOC at ${t}_{o}$ (0 min); $C$ (mg/L) is the concentration of the TOC at ${t}_{t}$ (min); ${k}_{1}$ (1/min) and ${k}_{2}$ (L/mg^**.**^min) are the rate constants of pseudo-first-order (Eq. (3)) and pseudo-second-order (Eq. (4)), respectively. The TOC initial concentrations are 4.98 mg/L and 16.43 mg/L for the River Nile sample and final effluent of WWTP, respectively.

**Table 1:** The parameters obtained from models of kinetics

Kinetic model	Kinetic parameter	River Nile	Effluent of WWTP
Pseudo-first-order	${k}_{1}$(1/min)	0.076 ± 0.009	0.138 ± 0.012
	*R*^2^	0.949	0.986
	*χ*^2^	0.005	0.002
	SSE	0.016	0.007
Pseudo-second-order	${k}_{2}$(L/mg^**.**^min)	0.011 ± 0.003	0.008 ± 0.002
	*R*^2^	0.909	0.945
	*χ*^2^	0.009	0.009
	SSE	0.028	0.026

An assessment of the error function is typically needed to determine whether a model equation is appropriate for use with experimental results. Error functions were utilized to calculate the differences between theoretical and experimental data. Utilizing the coefficient of determination (${R}^{2}$), chi-square (${\chi }^{2}$) test, and sum of square error (SSE) statistical error functions, the best-fitting of TOC degradation kinetics was verified. These error functions show that pseudo-first-order kinetics is fitted to the experimental data for both two water samples (Table 1), as it shows the lowest SSE, ${\chi }^{2}$, and the closest value of ${R}^{2}$ to unity. The same behavior was observed by El-Kalliny et al. (2021) with the degradation of crystal violet dye in aqueous media by CAP treatment. To sum up, the final effluent of WWTP shows a faster degradation rate by CAP treatment compared with the river Nile sample, and the TOC degradation kinetics can be described as pseudo-first-order.

**Fig. 4:**
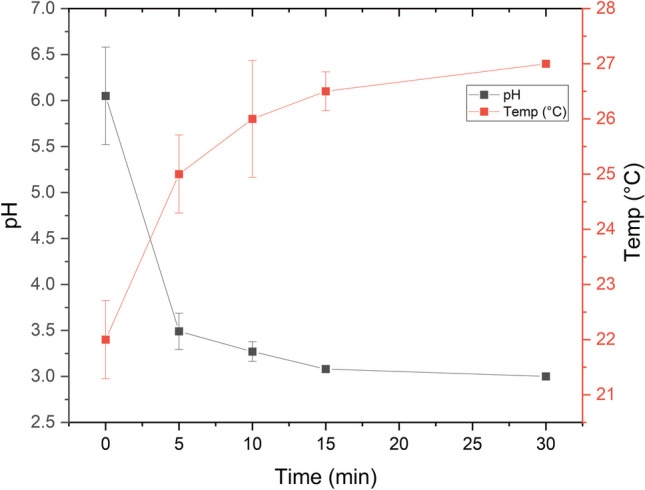
Impact of CAP treatment on the temperature and pH during the course of dH_2_O treatment

During CAP treatment, the pH of the aqueous solution changed. The pH of dH_2_O decreased, as shown in Fig. 4. The formation of nitrous and nitric acids in the plasma-treated fluids by the reaction of the electric charge with the nitrogen gas in the air was mostly responsible for the pH reduction (Lukes et al. 2012). However, the water matrix plays a role in the changing of the pH as well.

Also, Fig. 4 shows that temperature increases during CAP treatment are often limited. Temperature changes rely on a variety of parameters, such as plasma power, treatment duration, plasma-gas composition, water volume, and initial water temperature. Therefore, temperature monitoring and management are critical during CAP treatment to enable efficient treatment while preventing any unwanted thermal impacts on the treated water or its intended usage.

Table 2 presents the physicochemical properties of the two different water matrices before and after CAP treatment including the cationic and anionic species. CAP treatment leads to a significant increase in nitrites and nitrates in the treated water, and this is the main reason for decreasing the pH due to the formation of nitrous and nitric acids. However, the pH value slightly increased by CAP treatment for the WWTP final effluent. This can be attributed to the increase in the ammonia concentration which may be liberated by the CAP oxidation for N-containing organic compounds in the WW matrix. Generally, in both the Nile River and WWTP final effluent samples, the values of TDS increased by CAP treatment, and consequently the EC values increased. This is reflected in the increase of some cationic and anionic species in the treated water samples. This might be due to the concentration of the water samples through the non-equilibrium vaporization results from the bombardment of energetic ions from the solution surface when the plasma interacts with the water surface (Ekanayake et al. 2020). Accordingly, the CAP process is effective for the removal of organic pollutants, while it can cause an increase in some inorganic species in the final treated effluent.

**Table 2:** Characterization of the two different water matrices before and after CAP treatment

Parameter	Nile river	CAP-treated Nile river	WWTP final effluent	CAP-treated WWTP final effluent
pH	7.9	7.4	7.7	8.1
EC (µS/cm)	200	260	300	429
TDS (mg/L)	97	133	150	200
Total hardness (mg/L)	114.46	106.38	147.49	152.58
F^−^ (mg/L)	0.428	0.433	0.35	0.36
Cl^−^ (mg/L)	15.25	15.39	63.34	66.22
NO_2_^−^ (mg/L)	0.05	65.17	4.29	83.15
NO_3_^−^ (mg/L)	2.55	40.95	7.94	47.06
PO_4_^3−^ (mg/L)	0.14	0.01	1.05	1.13
SO_4_^2−^ (mg/L)	20.35	20.53	56.04	59.12
HCO_3_^−^ (mg/L)	146	52	160	88
NH_4_^+^ (mg/L)	0.03	0.01	10.63	13.05
Na^+^ (mg/L)	24.29	30.34	69.94	74.46
K^+^ (mg/L)	5.04	7.34	15.36	15.11
Mg^2+^ (mg/L)	9.89	9.63	13.63	14.03
Ca^2+^ (mg/L)	29.57	27.95	36.63	38.01

**Fig. 5:**
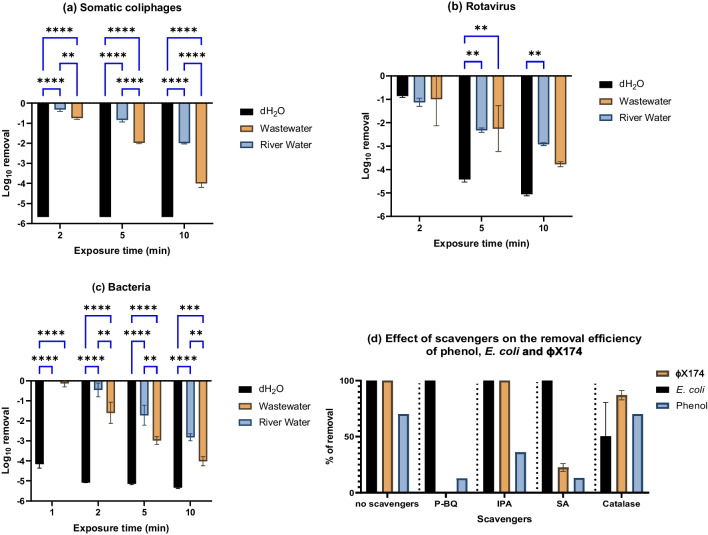
**a**, **b** Viral and **c** bacterial removal from river water and wastewater samples by CAP. The reactor diameter is 5.6 cm, and the experiments were conducted in a batch system. A two-way ANOVA with Tukey’s multiple comparisons test was conducted. Only comparisons with *p* ≤ 0.01 are displayed as asterisks. **d** Effect of scavengers on the removal efficiency of phenol, *E. coli* and ɸX174. All scavengers have a concentration of 100 mM except catalase (40 μg/10 mL). Data are an average of duplicate batch experiments, and the error bars indicate the SEM

The effect of CAP on viruses, bacteria, and protozoa was also investigated in river water and wastewater samples and compared to its performance in dH_2_O. In Fig. 5a, ~ 2log_10_ and 4log_10_ reductions of ɸX174 were obtained after 10 min of CAP exposure in river water and wastewater, respectively. Water matrices played a crucial role in the reduction of CAP efficacy because only 1 min was enough for the complete removal of ɸX174 from dH_2_O. Also, 5 min of CAP treatment was required for the complete removal of RoV from dH_2_O, whereas only ~ 2.0log_10_ was removed from river water and wastewater, at the same treatment time (Fig. 5b). The majority of viruses in surface water and wastewater are probably aggregated together or attached to organic materials, impairing the rates of viral inactivation (Kahler et al. 2016).

CAP can damage viral nucleic acids, leading to improper gene expression. The viral capsid proteins can get peroxidized and lose its infectivity when active species come into contact with it. Plasma can inactivate bacteriophages through a number of singlet oxygen-related mechanisms. Damage to viral DNA and proteins may result from viral DNA-DNA crosslinking or DNA–protein complexes caused by plasma exposure. Additionally, ^1^O_2_ was supposed to be the most efficient method for inactivating the bacteriophages T4 and feline calicivirus (FCV). Methionine molecules are altered, and histidine molecules are oxidized (Aboubakr et al. 2015). One way that ^1^O_2_ affects capsid proteins is by interfering with enzyme function when it reacts with certain amino acids (Guo et al. 2018). It can alter the nucleic acid and protein through the formation of cross-links between guanine and lysine as a result of the oxidation process.

Also, H_2_O_2_ has been shown to play a considerable role in the inactivation of enveloped viruses but only a minor effect on naked viruses (Sakudo et al. 2016). Considering that the current investigation employed naked viruses, it is anticipated that H_2_O_2_ will only have a slight effect on viral inactivation.

The inactivation of ɸX174 after brief contact with plasma might be attributable to viral capsid destruction or conformational change (Yasuda et al. 2010). It was reported that the role of plasma on bacteriophage lambda was predominantly caused by damage to the protein coat and, to a lesser extent, by damage to viral DNA. Whereas, if the virus is exposed to plasma for an extended period of time, both DNA and protein can be destroyed (Yasuda et al. 2010), and causing bacteriophage aggregations (Davies 2016). A comparable route of CAP inactivation was identified for bacteriophage MS2 and adenovirus (Zimmermann et al. 2011). The effect of CAP treatment on viral nucleic acid is presented in Table (3).

Data showed that lower action of RoV inactivation in the present study was attributed to the change in viral nucleic acid which was not increased with longer CAP exposure time, as indicated by direct RT-qPCR. RT-PCR analysis of a longer portion of the viral genome to assess genomic integrity can provide a stronger correlation with virus infectivity (Hamza et al. 2011). Also, damage to viral capsid proteins is possible, as estimated by ICC-RT-qPCR in which infectivity was lost, but minimal reduction (<0.5log_10_) by direct RT-qPCR at the same exposure time was observed (Table 3). This data is consistent with Aboubakr et al. (2018) and Yasuda et al. (2010), who demonstrated that during CAP treatment of bacteriophages and FCV, the damage of capsid proteins precedes the degradation of nucleic acids. In contrast, degradation of viral nucleic acid was the main effect of CAP treatment on FCV (Yamashiro et al. 2018), potato virus *Y* (Filipić et al. 2019) and adenovirus (Sakudo et al. 2016).

**Table 3:** The effect of CAP treatment on viral nucleic acid and infectivity

Treatment time (min)	Direct RT-qPCR		ICC-RT-qPCR	
	Log (*N*/*N*_0_)	% removal	Log (*N*/*N*_0_)	% removal
1	− 0.11	22.39	− 0.31	50.50
2	− 0.37	57.37	− 0.90	87.41
3	− 0.36	56.17	− 2.51	99.69
5	− 0.41	61.39	− 3.06	99.91
10	− 0.43	63.0	− 6.18	100.00

Similarly, *E. coli* counts were reduced by 3log_10_ and 4 log_10_ after 10 min of CAP treatment of river and wastewater samples, respectively. On the other hand, 4 log_10_ removal of *E. coli* was achieved in dH_2_O after 30 s of contact time (Fig. 5c). In another study, *E. coli* was reduced by 3log_10_ removal in wastewater effluent using CAP (Liao et al. 2021). According to Van Nguyen et al. (2019), CAP reduced the concentration of *E. coli* in river water samples by 99.8% for 15 kV and completely removed it for 18 kV. The given results support the conclusion that CAP has significant anti-bacterial properties due to the diffusion of RONS through the air gap and dissolving into the aqueous solution (Hefny and Tawfik 2022; Tawfik and Hefny 2021). CAP has ability to destroy the bacterial cell membranes of *E. coli* and damage the intracellular composition of bacterial cells such as bacterial nucleic acids and enzymes (Liao et al. 2021).

Peroxynitrite has been identified as one of the major reactive species generated by CAP and inactivate *E. coli* (Zhou et al. 2018). Su and Groves (2010) calculated the permeability coefficient for peroxynitrite to be 8.0 × 10^4^ cm/s, which is comparable to that of H_2_O_2_ but about 400 times larger than that of superoxide. Because of its great permeability, peroxynitrite is particularly a potent oxidant for causing bacterial damage. It was shown that short exposure to CAP causes an instant loss of cell membrane integrity (Abdel-Wahed et al. 2022).

Using *E. coli* and total coliform, Nguyen et al. (2019) reported that CAP could be combined with conventional surface water pre-treatment methods for water disinfection. Interestingly, after treatment with CAP, all metrics of surface water met the acceptable criteria for drinking water quality.

The CAP system produces a variety of radicals of ROS and RNS with different levels depending on the type and composition of the water matrix. As a result, and for the sake of simplicity, radical scavengers can be used to estimate the contribution of the majority of generated radicals rather than tracking the concentration of these radicals. Distilled water is a good water matrix in terms of generalization to be a control for different water matrices. Figure 5d shows the role of the main reactive species O_2_^·^, ^1^O_2_, ^·^OH, and H_2_O_2_, produced in the CAP process into the chemical and microbial removal. In this Figure, instead of measuring TOC, the phenol was employed as a model water pollutant to eliminate any interference from the organic scavengers. Para-benzoquinone (p-BQ), sodium azide (SA), isopropyl alcohol (IPA), and catalase scavengers can trap effectively the reactive species O_2_^·^, ^1^O_2_, ^·^OH, and H_2_O_2_, respectively (Abdel-Wahed et al. 2022; Bekeschus et al. 2017; Santos et al. 2012). The experimental conditions of trapping are indicated in the Figure caption.

After adding p-BQ, IPA, and SA, the percentage of phenol removed dropped, indicating that O_2_^·^, ^1^O_2_, and ^·^OH play a significant role in the degradation of phenol by CAP. The contribution of oxidizing species in the phenol degradation process occurs in the following order: O_2_^·^ = ^1^O_2_ > ^·^OH. H_2_O_2_ had no effect, since adding catalase had no influence on the amount of phenol removed. Although, the virucidal impact (Fig. 5a, b) may result from the chemical reactions between the viral components and RONS like ^1^O_2_, O_3_, ${\mathrm{O}}_{2}^{\cdot -}$, and peroxynitrous acid, Fig. 5d shows that ^1^O_2_ has a substantial role in ɸX174 inactivation, as evidenced by the observed reduction in CAP performance to inactivate ɸX174 in the presence of SA; whereas, H_2_O_2_ has a significant a role in the reduction of *E. coli* in the treated samples.

Actually, there is a research gap on the effect of CAP on protozoa. *Acanthamoeba* was selected as a protozoan pathogen model to investigate the ability of CAP to disinfect its resistant cysts in different water matrices. This pathogen is characterized by a hard cyst wall, which can resist extreme environmental conditions and physical and chemical disinfectant doses such as chlorine, UV, and ozone (Dupuy et al. 2014; Hijnen et al. 2006; Thomas et al. 2004). Moreover, *Acanthamoeba* spp. is easy to cultivate using economic media (non-nutrient agar seeded with *E. coli*).

**Fig. 6:**
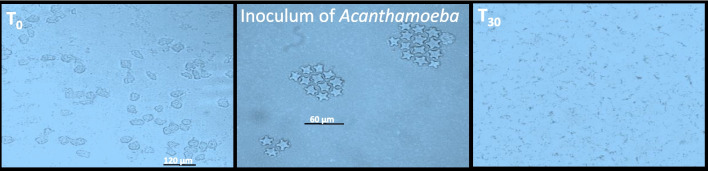
The effect of CAP treatment on *Acanthamoeba* in dH_2_O. T_0_ shows cultivation of a spiked water sample without treatment. T_30_ represents the cultivation of a spiked sample after 30 min of CAP treatment showing only bacterial growth. Plates have been incubated for 1 week and examined daily for the presence of *Acanthamoeba* trophozoites or cysts

Data in Fig. 6 indicate that CAP has the potential to treat *Acanthamoeba* in water. However, it is clear that water matrices have a negative impact on the performance of CAP to remove protozoa; as in the case of dH_2_O, complete inactivation was obtained after 30-min treatment, while no removal of *Acanthamoeba* was observed after 30 min of exposure in river or wastewater samples (Fig. 6). Additionally, *Acanthamoeba* inactivated by CAP in dH_2_O showed negative PCR signals indicating degradation of *Acanthamoeba* nucleic acid.

This means that the assay still needs more optimization in order to achieve complete removal of protozoa from complex water matrix. However, it is well established that *Acanthamoeba* is highly resistant to water disinfectants. Due to the limitations of the *Acanthamoeba* detection technique which involve the direct cultivation of the treated samples, *Acanthamoeba* might be reduced in environmental water samples by CAP treatment, but log_10_ reduction could not be measured after cultivation and cyst generation of *Acanthamoeba*. Therefore, the detection of *Acanthamoeba* in treated water samples was based on qualitative not quantitative method. The same experiments have been conducted using *Nagleria clarki*, which showed lower resistance to CAP compared to *Acanthamoeba*, represented by complete inactivation in 15 min treatment (data not shown).

The effect of CAP has been investigated before on another type of protozoa (*Cryptosporidium parvum*) oocysts (Craighead et al. 2020), revealing that oocysts were inactivated by 2.0 log_10_ after 3 min of treatment by CAP supporting the idea of the potential use of CAP to treat protozoa in water. This is a promising development in the field of water treatment, as protozoa can be harmful to human health if ingested and are more resistant to disinfectants compared to viruses and bacteria. 

CAP technology may offer a more efficient and cost-effective solution compared to traditional water treatment methods. The difference between the microbial removal by CAP in dH_2_O and environmental water samples could be due to the presence of organic matter in environmental water which can directly interact with active species, reducing the biocidal activity of CAP. Therefore, it is important to consider the composition of the treated water and the potential impact of organic and inorganic materials on the effectiveness of microbial removal.

Barillas (2015) designed a prototype CAP reactor to be used a tertiary treatment of the industrial wastewater. One of the primary advantages of scaling up plasma devices for field applications is that the size of the plasma-liquid interface may be expanded allowing for the exposure of thin liquid layers to CAP which could enhance the efficacy of the treatment (Foster et al. 2018).

## Conclusion

Water plasma treatment is an innovative approach to generating physiologically active solutions that may not be readily generated using chemical methods alone. As such, this method warrants additional research and development as a green stand-alone alternative or supplementary approach to traditional water treatment techniques. The plasma voltage and current waveforms were measured during the treatment, and the plasma power and the consumed energy were calculated as well. Moreover, the OES during plasma treatment of dH_2_O were measured and analyzed, and the electron density was estimated from the OES. CAP was examined for its effects on microbial and chemical pollutants in different types of water. Water type affected the efficacy of CAP treatment. For example, 1 min of exposure was enough to complete removal of ɸX174 in dH_2_O, whereas a 10-min application of CAP reduced ɸX174 by 2 log_10_ in river water and 4 log_10_ in wastewater. In the case of RoV, 5-min CAP treatment were required for its complete removal in dH_2_O and for ~ 2log_10_ in river water and wastewater. Similarly, *E. coli* reduced by 3log_10_ and 4log_10_ after 10-min treatment in river water or wastewater and 30 s achieved 4log10 *E. coli* reduction in dH_2_O.

The approach successfully reduced TOC from both surface and wastewater samples. TOC degradation kinetics may be represented by pseudo-first-order, and the final effluent of the WWTP exhibits a higher rate of CAP treatment degradation compared to the river Nile sample. According to the study, in the CAP process, singlet oxygen and superoxide radicals both have significant equal contributions to the phenol degradation. In addition, the CAP method removes organic contaminants effectively, but it also results in an increase in inorganic species in the final treated effluent. The data presented here support the potential combination of CAP with conventional pretreatment steps for water and wastewater purification. Indeed, comparisons of different studies of CAP are problematic due to the use of varied plasma system configurations. As a result, it is critical for researchers to develop a standardized process for evaluating and comparing CAP systems to assure accurate and consistent results. This will allow for the creation of more effective and efficient plasma-based technologies for a variety of applications. Further work is needed to fully understand the effectiveness and safety of this technology. In terms of cost, traditional procedures such as chlorination and UV irradiation have lower operational expenses than CAP. However, CAP is a newer technology that is still being optimized, and a cost-effective CAP system could accelerate the progress of the technology and become more commonly used.

## Data Availability

All the data generated or analyzed during this study are included in this published article.
